# Transient and Permanent Experience with Fatty Acids Changes *Drosophila melanogaster* Preference and Fitness

**DOI:** 10.1371/journal.pone.0092352

**Published:** 2014-03-25

**Authors:** Justin Flaven-Pouchon, Thibault Garcia, Dehbia Abed-Vieillard, Jean-Pierre Farine, Jean-François Ferveur, Claude Everaerts

**Affiliations:** Centre des Sciences du Goût et de l’Alimentation, UMR 6265 CNRS, UMR 1324 INRA, Université de Bourgogne, Dijon, France; Institut de Génomique Fonctionnelle, France

## Abstract

Food and host-preference relies on genetic adaptation and sensory experience. In vertebrates, experience with food-related cues during early development can change adult preference. This is also true in holometabolous insects, which undergo a drastic nervous system remodelling during their complete metamorphosis, but remains uncertain in *Drosophila melanogaster*. We have conditioned *D. melanogaster* with oleic (C18∶1) and stearic (C18∶0) acids, two common dietary fatty acids, respectively preferred by larvae and adult. Wild-type individuals exposed either during a transient period of development–from embryo to adult–or more permanently–during one to ten generation cycles–were affected by such conditioning. In particular, the oviposition preference of females exposed to each fatty acid during larval development was affected without cross-effect indicating the specificity of each substance. Permanent exposure to each fatty acid also drastically changed oviposition preference as well as major fitness traits (development duration, sex-ratio, fecundity, adult lethality). This suggests that *D. melanogaster* ability to adapt to new food sources is determined by its genetic and sensory plasticity both of which may explain the success of this generalist-diet species.

## Introduction

Animal diet can be specialized or generalist. In a novel environment, animal adaptation to the available food resources mostly depends on two biological processes – genetic adaptation and individual sensory experience – acting on different time scales [1], [2]. The genetic network underlying the physiological ability to assimilate nutrients contained in the novel food depends on a process of natural selection requiring a variable number of generations [3]. The selection of genetic alleles changing adaptation and fitness to novel food has been described in various animals and in humans [4], [5]. Differently, individual sensory experience occurs during a shorter period of time: a transient exposure to food-related stimuli during early development can change the response of exposed animals to these stimuli. This effect was described in humans and rodents exposed to food-related compounds during their foetal development or prenatal life [6], [7] and in holometabolous insects (with a complete metamorphosis: Coleoptera, Lepidoptera, Diptera) encountering a drastic remodelling of their nervous system [8], [9], [10]. Some of these insects, pre-exposed to menthol during larval development, showed an altered adult response to this molecule [11]. However, this effect – also described in *Drosophila melanogaster –* was criticised from a methodological point of view [12]. In this species, chronic food deprivation during larval development can affect adult behaviour and fitness [13]. Sensory conditioning during early adult development (less than one day-old) can also affect behaviour [14], [15], [16].

In this study, we exposed Drosophila to dietary fatty acids (FAs) which are important or essential nutrients in the diet of most – if not all – animals [17], [18]. Essential FAs cannot be synthesized (as ω-6 FAs in human) and they play a critical role in varied functions such as reproduction, cold adaptation, metabolism and cell signalling both in mammals and insects [18]. However, their overconsumption (mostly of saturated FAs) has dramatic health consequences (high blood pressure, deregulated glycaemia, dyslipidemia, obesity) and is though to increase the risk of some cancers [19], [20], [21]. Defects associated with FA-exposure can be transmitted to the progeny: a high proportion of saturated FAs in the diet of the pregnant mother statistically increases the risk of metabolic syndrome and of brain alteration in her offspring [18]. Some of these deleterious effects are induced by the epigenetic effect of FAs modifying gene expression during early foetal development [22], [23]. Some insects also detect and prefer FAs: the adult mosquitoes *Aedes aegypti* and *Anopheles gambia*, and the nymphal bug *Triatoma infestans* are attracted by specific FAs (combined with L-lactic acid) secreted by the human skin [24], [25], [26]. However, adult mosquitoes and flies can also be repulsed by some FAs tested either alone or combined with volatile substances [27], [28]. The preference of *D. melanogaster* to pure dietary FAs changes during life: larvae prefer unsaturated FAs whereas adults prefer saturated FAs [29]. However, the adaptative value of this developmental shift remains unclear.

**Figure 1:**
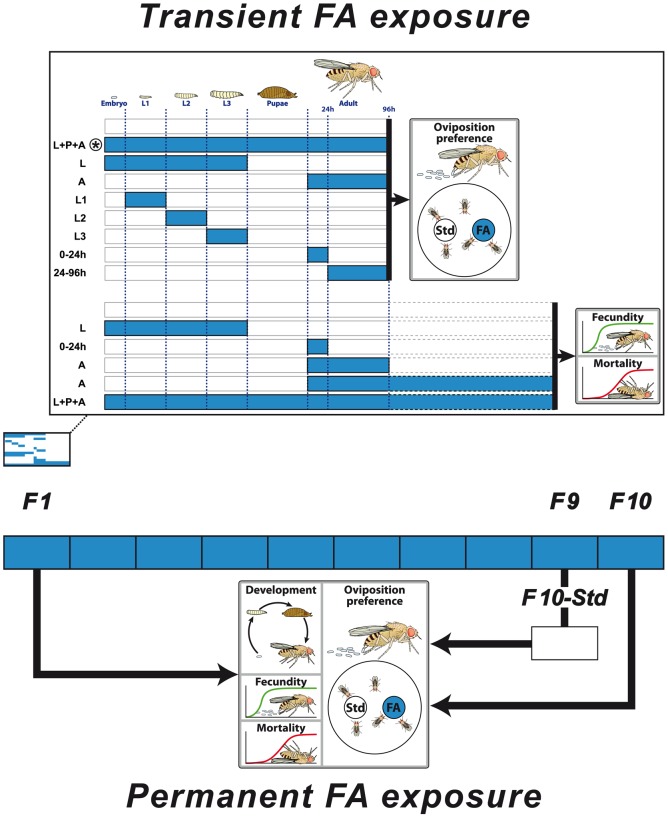
Experimental procedures. Representation of the schematic procedure used for transient exposure (top) and permanent exposure (bottom) to fatty acids and the principal phenotypes to measure the effect of exposure (Oviposition preference in a dual-choice test; Fecundity; Adult mortality; Development duration). The blue colour represent the exposure period on the fatty acid (FA); the white colour is the period on standard food (Std). Transient exposure was performed during different developmental phases (indicated on the left side; see also Material and methods; E = embryo, L1, L2 & L3 = 1^st^, 2^nd^ & 3^rd^ larval instar). * corresponds to the exposure period used for the cross-conditioned experiment. For permanent exposure, individuals were either (*i*) kept on the FA during one generation (F1), (*ii*) 10 generations (F10), or (*iii*) 9 generations and returned on standard food for the 10^th^ generation (F10-Std).

To test the possible effect of FA conditioning on developmental preference, we exposed *D. melanogaster* to two pure dietary FAs, stearic (C18∶0) and oleic acids (C18∶1) which are very common in natural food. Both FAs have a similar carbon chain and diverge by only one unsaturation. Exposure occurred either during a single developmental period (from larval to imaginal life) or during one to ten complete generations (Figure 1). We measured the consequence of exposure on oviposition preference and fitness (development duration, sex-ratio, fecundity, larval size, and larval and adult survival).

## Materials and Methods

### Flies

We used a wild-type strain of *D. melanogaster* Meigen, Dijon 2000 (Di2) – maintained in our lab for more than a decade and which showed very stable behavioural performances. Flies were raised in 150 ml glass vials containing 50 ml of yeast/cornmeal/agar medium and kept in a breeding room at 24.5±0.5°C with 65±5% humidity on a 12∶12 h light/dark cycle. Flies were transferred every two/three days to avoid larval competition and to regularly provide abundant progeny for testing. All exposures and experiments were performed under similar conditions.

### Food

Stearic (C18∶0) and oleic (C18∶1; both from Sigma-Aldrich) acids were kept at −20°C. FA-rich media were prepared with 500 mg of either FA diluted in 500 μL EtOH and mixed with 100 mL warmed-up (60°C) standard medium to obtain the 5 μg/μl final concentration. The same EtOH volume was added to make the control medium (standard). Using gas-chromatography, we determined the FA content of standard medium (0.004±0.002 μg/μl C18∶0; 0.016±0.007 μg/μl C18∶1). These amounts correspond to 0.08 and 0.32% of the FA content in the C18∶0- and C18∶1-rich media. All media were freshly prepared in our lab and used within one week.

### Behaviour

#### Exposure protocols

*TRANSIENT EXPOSURE*: For all exposure periods, emerging flies were sexed 0–4 hour after emergence under light CO_2_ anaesthesia and kept by groups of 10 (females) or 20 (males) until the test. Flies were exposed to FA during 8 distinct periods: (1) early embryo to adult 96 hours old (L+P+A), (2) all larval development (L), (3) all adult life until 96 hours old (A), (4) 1^st^ larval instar (L1), (5) 2^nd^ larval instar (L2), (6) 3^rd^ larval instar (L3), (7) early adult life until 24-hours-old (0–24 h) and (8) adult life between 24 and 96 hours old (24–96 h).

For L+P+A- and L-exposures, 50 Di2 eggs were deposited in vials containing either C18∶0- or C18∶1-rich media. L+P+A exposed adult males and females were kept on FA-rich medium. For L-exposure, pupae were transferred onto standard food and emerging flies maintained on the same medium. For A-exposure, Di2 flies collected at emergence were kept during 4 days in vials containing either C18∶0-, or C18∶1-rich food. For L1-, L2- and L3-exposures, 50 Di2 eggs were deposited on standard medium. For staging, larvae were screened under a binocular microscope and collected according to a clear morphological marker (spiracles shape [30]). Early L1, L2 or L3 were transferred from standard food to FA-rich medium during 24 h, then carefully cleaned and brushed with distilled water, and returned on standard medium.

For «0–24 h» and «24–96 h» exposures, emerging Di2 adults were housed in same-sex group on FA-enriched or standard media, respectively. After 24 h, FA-exposed sex-mixed adults were returned to standard medium until they were 96 hours old. 24–96 h-adults to be exposed were transferred from standard food to FA-enriched medium when 24-hour-old.

*PERMANENT EXPOSURE:* For permanent exposure, 50–100 Di2 eggs were deposited in vials containing either C18∶0- or C18∶1-rich media and resulting individuals were raised on FA-enriched medium during one to 10 generations (10 vials were kept at each generation). The effect of permanent exposure to FAs was measured on oviposition preference, developmental time, fecundity, larval and adult mortality during the first generation cycle (F1) and after 10 generations (F10). Some individuals were kept 9 generations on FA and then transferred for an extra generation on standard food (F10-Std). The performance of these lines was compared to that of a control (naïve) strain simultaneously raised.

#### Oviposition test

After mass-mating, five 4-day-old female flies were immediately introduced into a glass container (Duran, 95 mm diameter, 100 mm high with a transparent lid) containing two Petri dishes (Greiner bio-one, 35 mm diameter, 10 mm high) filled with either FA-rich or standard media. After, 20 hours, the number of eggs on each egg-laying area was counted to determine the oviposition preference index (OPI) as follow:
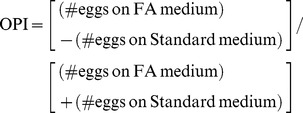



#### Fitness parameters

*DEVELOPMENTAL TIME:* 50 Eggs were deposited in plastic vials either on standard medium or FA-rich medium. After 24 h, hatched eggs were counted. Thereafter, white pupae and emerging male and female adults were counted every 24 h.

*FECUNDITY:* Adult flies, sexed at emergence, were housed by 10 (females) or 20 (males) on FA-rich or standard media. After mating, 4-day-old female flies were individually introduced and transferred every 24 h into a new glass vial filled with the FA-rich or standard media. The number of eggs (total, unfertilized and non-viable) was counted after 48 h. Conditioning did not significantly affect the fertilized/unfertilized eggs ratio (data not shown).

*LIFESPAN:* Flies were sexed at emergence and kept in same-sex groups of 20 in glass vials containing either FA-rich or control media. Every 48 h, flies were transferred in fresh vials filled with a similar medium and the dead flies counted.

### Statistical Analysis

Statistical analyses were conducted with XLSTAT 2012.1.01 [31] and Microsoft Excel 14.2 (MacOS 10.8.4). The *Development Times 50* (DT_50_) and *Lethal Times 50* (LT_50_) were computed using Probit regression. OPI, DT_50_, the *Cumulative Number of Eggs*, the *Cumulative Mortality at day 22* and the *Sex-Ratio* were compared with the Kruskal-Wallis test, completed by Conover-Iman’s multiple pairwise comparison (two-tailed with Bonferroni correction). To assess female fecundity of F1, F10 and F10-Std females, we also conducted an analysis of covariance using «*egg number*» as quantitative dependant variable, «*time after the egg-laying onset*» as explanatory quantitative variable and «*exposure X test*» as explanatory qualitative variable. This ANCOVA was completed by a Fisher LSD multiple pairwise comparison of factors.

Except for fecundity, data were shown as box-plot (indicating 1^st^ quartile, median and 3^rd^ quartile; the «whiskers» indicates the 95% limits), with mean (open circles) and SEM (error bars).

## Results

**Figure 2:**
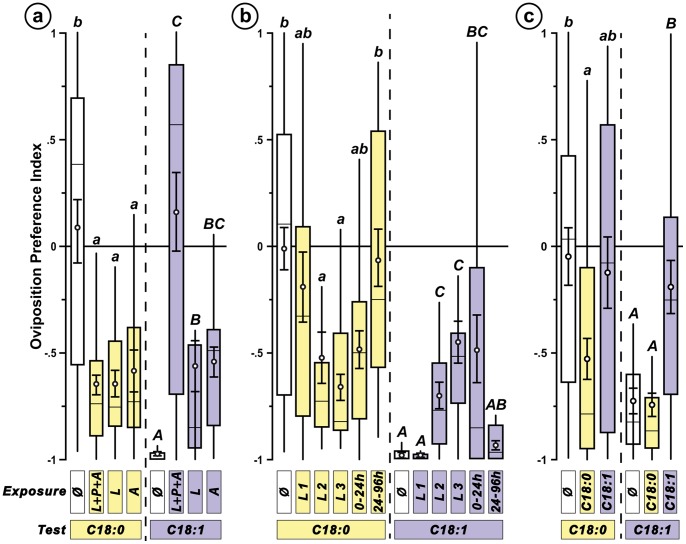
Exposure to fatty acids during critical periods specifically changes oviposition behaviour. Coloured bars indicate the distribution of oviposition response in females tested to each fatty acid (Test: C18∶0 = yellow; C18∶1 = purple) after various exposures to these substances (Exposure: same colour code, under the bars). White bars indicate the response of control naïve lines exposed to standard food and tested simultaneously with experimental lines. Exposure periods correspond to (**a**) larval+pupal+adult development (L+P+A), larval development (L), or adult life (A), to (**b**) each larval stage (L1, L2, L3), or two periods of adult life (0–24 h, 24–96 h). For cross-conditioning experiment (**c**), «L+P+A-exposed» individuals were tested to both FAs. Data are represented both with their mean (open circles) ± SEM (error bars) and by box-and-whisker plots (the bars represent the first and third quartiles (Q1 and Q3) with the horizontal line inside the bar indicating the median value; the whiskers (vertical line on both sides of the bar) indicate the limits beyond which values are considered anomalous – limits were calculated as follows: lower limit = Q1–1.5×[Q3– Q1] and upper limit = Q1 = 1.5×[Q3– Q1]). For each panel, differences were assessed with a Kruskal-Wallis test (letters indicate significant differences at level *p* = 0.05). *N* = 18–20 (a), 18–37 (b), 22–28 (c).

Our goal consisted to measure the behavioural and fitness consequences of a transient or a permanent exposure to C18∶0 or to C18∶1 (Figure 1). First, we determined the effect of a transient exposure, during preimaginal and/or early imaginal development, on oviposition preference. Exposure either took place (1) from embryonic to 4-day-old adult life ( = larval+pupal+adult development; L+P+A), (2) during all larval development (L), or (3) during the four first days of adult development (A = 0 to 96-hour-old adult life; Figure 2a). All exposures with the two FAs clearly affected oviposition behaviour. All females exposed to C18∶0 showed a strong aversion to this substance compared to naïve indifferent females (Kruskal-Wallis test: KW_3df_ = 12.68; *p* = 0.005). Reciprocally, females exposed to C18∶1 were less repulsed by this substance than naïve females; in particular «L+P+A» exposure induced an indifferent response (KW_3df_ = 29.27; *p*<0.0001). This clearly indicates that the exposure to each FA either during larval and/or imaginal development can change adult response to the encountered FA.

To better delimitate the critical period during which FA exposure affects preference, we restricted the duration of exposure either during each larval stage (L1, L2, L3), or during two periods of early adult development (0–24 h, 24–96 h; Figure 2b). L2 or L3 exposure to C18∶0 clearly induced adult aversion to this substance (KW_5df_ = 20.99; *p* = 0.001). Exposure to C18∶1 during L2, L3 stages or early adult life (0–24 h) strongly reduced the strong aversive effect normally induced by this substance in naïve flies (KW_5df_ = 48.82; *p*<0.0001). These experiments reveal that transient exposure to each FA during preimaginal development affects adult response. Early imaginal exposure to C18∶1 also affected adult preference.

Given the great similarity of the effect induced by the conditioning with each FA, we tested their specificity. Individuals were «cross-conditioned» to a given FA («L+P+A» exposure) and their response was measured with the other one. C18∶0 exposure did not affect adult response to C18∶1 (Figure 2c; KW_2df_ = 7.01, *p* = 0.030). Note that C18∶0-conditioned females showed a reduced, although still significant, repulsion against C18∶0 likely because individuals were exposed during a longer developmental period if compared to the previous experiments (Figure 2a, b). Similarly, females conditioned with C18∶1 showed no changed preference to C18∶0 (KW_2df_ = 17.23, *p*<0.0001). Therefore, conditioning individuals with each FA induced a specific effect on their oviposition preference.

We also measured two fitness traits – fecundity and adult viability – in transiently exposed individuals. Fecundity (counted as the number of fertilized eggs per female) was drastically reduced in naïve flies and «L+P+A» C18∶1-exposed flies kept on C18∶1 medium (Figure S1a; KW_10df_ = 67.26; *p*<0.0001). The only significant effect on mortality was induced by 0–96 h exposure to each FA which reduced male lifespan (Figure S1b, c; KW_6df_ = 40.08; *p*<0.0001).

**Figure 3:**
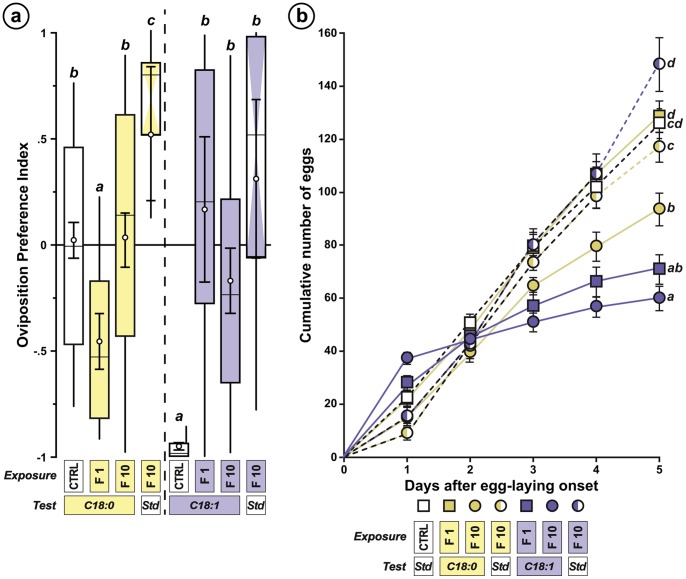
Effect of permanent exposure on oviposition preference and fecundity. Oviposition preference (a) and fecundity (b) of lines exposed to FA-rich food during one generation (F1), 10 generations (F10), or 9 generations+the 10^th^ generation on standard food (F10-Std). (**a**) For oviposition response, see legend of Figure 2 (*N* = 10–25). (**b**) Fecundity was measured in flies (females and males mixed) similarly exposed. The daily number of eggs (noted between days 1 to 5; corresponding to the female age) was cumulated. Letters indicate significant differences (*p* = 0.05) for the «*exposure X test*» variable (ANCOVA; F_(7,517df)_ = 137.52; *p*<0.0001; *N* = 14–16).

Next, we measured the effect of permanent exposure to each FA during one or 10 complete generation cycles (F1 and F10, respectively; Figure 3). To distinguish genetic adaptation (between F1 and F10) and environmental effects (directly caused by FA exposure), we also measured the response of individuals, kept for 9 generations on FA and transferred during the 10^th^ generation on standard food (F10-Std). Concerning oviposition response, C18∶0 exposure induced a non-durable effect (F1 but not F10 individuals significantly changed their response; KW_3df_ = 14.19; *p* = 0.003; Figure 3a). Moreover, F10-Std females were slightly attracted to C18∶0. C18∶1 exposure induced a reduced aversion to C18∶1 (close to indifference) which remained constant after flies were shifted back on standard food (F10-Std; KW_3df_ = 36.60; *p*<0.0001).

Permanent F1 and F10 exposure to each FA also altered fitness traits such as fecundity, embryonic survival, embryo-to-adult time to develop, larval size and survival, sex-ratio and adult survival. Fecundity varied with FA diet: the number of fertilized eggs laid by F1 and F10 females kept on C18∶1 and that of F10 females kept on C18∶0 was lower than in controls (Figure 3b). This effect disappeared in both F10-Std lines (ANCOVA; F_(7,517df)_ = 137.52; *p*<0.0001). The proportion of hatched eggs increased in F1 and F10 – but not in F10-Std – C18∶0-exposed females compared to naïve and C18∶1-exposed females (Figure S2a; KW_6df_ = 14.09; *p = *0.03). Larval survival was not affected by exposure (Figure S2b).

**Figure 4:**
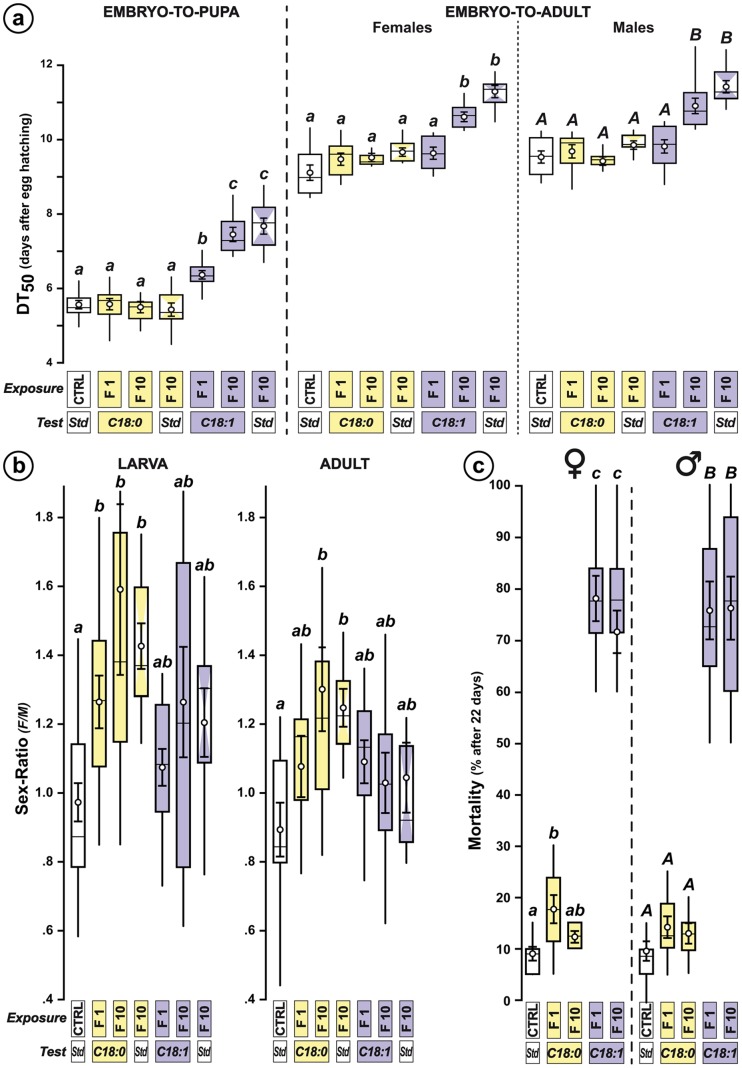
Effect of permanent exposure on preimaginal development, sex-ratio and adult lifespan. (**a**) The box-and-whisker plots show the Developmental Time 50 (DT_50_; computed on groups of 50 individuals), corresponding to the period of time required for 50% individual embryos to reach either pupation (embryo-to-pupa; left) or adult eclosion (embryo-to-adult) in females (center) and males (right; *N* = 10/experiment). (**b**) The plots represent the sex-ratio (females/males) in third instar larvae (Larva, left) and in adult progeny (Adult, right) of exposed lines (*N* = 9–25). (**c**) The plots indicate the cumulative mortality after 22 days of adult life in females (left) and males (right; *N* = 10/experiment). For all experiments, the exposure and test conditions are indicated below each graph. For more information and statistics, refer to Figure 2.

Exposure to C18∶1 increased the «embryo-to-pupa» developmental duration in all lines (KW_6df_ = 51.06; *p*<0.0001; Figure 4a) and the «embryo-to-adult» duration (+2 days) in both F10 females and males (KW_6df_ = 44.27 – *p*<0.0001 – and KW_6df_ = 45.00 – *p*<0.0001, respectively; Figure 4b). The size of C18∶1-exposed male and female larvae also increased (+30%) in both F10 and F10-Std lines (Figure S2c; KW_6df_ = 58.50 – *p*<0.0001 – and KW_6df_ = 80.00 – *p*<0.0001, respectively). Differently, C18∶0 exposure did not affect developmental duration and only slightly changed larval size.

Remarkably, exposure to C18∶0 rapidly (F1) and durably (F10, F10-Std) increased the sex ratio (female/male) of the larval and adult progeny (Figure 4b; KW_6df_ = 12.79 – *p* = 0.047 – and KW_6df_ = 22.13 – *p* = 0.001, respectively) whereas C18∶1 had no such effect.

Adult lifespan decreased in C18∶1-exposed F1 and F10 lines (Figure 4c; females: KW_6df_ = 12.79, *p*<0.0001; males: KW_6df_ = 40.08, *p*<0.0001; Figure S3). Differently, C18∶0 exposure only slightly altered F1 female viability.

## Discussion

Genetic and sensory adaptation to environment can influence life-history traits in vertebrates and invertebrates [32], [33], [34], [35], [36]. In insects, embryonic and larval experience can affect adult feeding [37], [38], [39], [40] and oviposition preference [41], [42], [43], [44]. Our data reveal that transient and permanent exposure to food molecules can change *D. melanogaster* food oviposition preference and fitness. In this species, the basis of chemosensory learning is well known in adult and larva [45], [46], but the effect of preimaginal conditioning on adult behaviour remains a matter of debate [12], [47], [48]. If several studies reported that larval exposure to diverse cues affects adult response [49], [50], [51], it was argued that chemosensory conditioning could «imprint» the young emerging adult during its physical contact with chemicals impregnating the pupal case. Hence, such contact could induce an «early imaginal exposure» affecting adult preference and behaviour [12], [52], [53], [54], [55]. Here, we show that conditioning *D. melanogaster* individuals with C18∶0 or C18∶1 during clear-cut developmental periods (at pre/imaginal stages) significantly altered their fitness and adult preference behaviour.

*D. melanogaster* shows different FA preferences during development: larvae prefer unsaturated FAs (C18∶1) whereas adults prefer saturated FAs (C18∶0) [29]. This developmental shift of preference could reflect stage-specific nutritional requirements. This may also reflect the different involvement of the sensory modalities (taste, olfaction and possibly mechanosensation) used during development for FA detection [18], [29]. The choice of the oviposition site by females is likely mediated by various sensory organs borne by the legs and the ovipositor [56], [57]. This behaviour is crucial, especially for her progeny [58] and our data suggest that preimaginal conditioning of individuals to novel food molecule may create a (associative or non-associative) learning effect. In particular, C18∶0 normally induces an «indifferent» response in naïve adults and is stressful for larvae [29]. However, when larvae are conditioned with C18∶0, they may associate this substance with stress, this explaining why exposed adult females showed an increased avoidance to C18∶0 (Figure 2). Reciprocally, naïve females avoid to lay eggs on C18∶1. However, since this FA, highly preferred by larvae, induced no developmental defect (at least in the F1 line), preimaginal exposure to C18∶1 may create a positive association of this FA with its «hedonic» value (maybe linking C18∶1 sensory perception and ingestion). Such positive association could explain the reduced aversion of exposed adults in our study (Figure 2). The substantial amplitude of the effect resulting of a transient exposure to these food molecules suggests that *D. melanogaster* possess a very high potential to adapt to novel diets.

When natural populations or species remain exposed for many generations to a novel diet, the genes underlying their food preference and fitness may permit adaption to this novel food source. After 10 generations, permanently exposed lines showed FA-specific behavioural and fitness changes some of which lasted after shift back on standard food (Table 1). For instance, on C18∶0-rich food, the proportion of adult females (relatively to adult males) increased after one generation and this bias persisted in F10 and F10-Std lines. Since no increased preimaginal mortality was noted, C18∶0-conditioning may increase female birth instead of male death. If this is true, it means that exposure to C18∶0 enhances fitness. Moreover, the persistence of this highly female-biased sex-ratio in the F10-Std line suggests that this effect has a genetic basis. Differently, the fact that embryonic viability increased in F1 and F10 lines, but not in F10-Std lines, suggests a direct (positive) effect of C18∶0 on viability. Finally, the observation that F10 lines showed rescued larval viability and female lifespan (which were altered in F1 lines) suggests that the genes underlying these phenotypes permit a quick adaption on C18∶0-rich food.

**Table 1:** Summary of the behavioural and fitness effects induced by permanent exposure to C18∶0 and C8∶1.

		C18∶0 exposed lines			C18∶1 exposed lines		
		F1	F10	F10-Std	F1	F10	F10-Std
		**OVIPOSITION PREFERENCE**					
		**−**	** = **	**+**	**+**	**+**	**+**
		**FITNESS**					
**LARVA**	Embryonic viability	**+**	**+**	** = **	** = **	** = **	** = **
	Larval viability	**−**	** = **	** = **	** = **	** = **	** = **
	Female larval size	** = **	** = **	**+**	** = **	**+**	**+**
	Male larval size	**+**	** = **	** = **	** = **	**+**	**+**
	Preimaginal develop. duration	** = **	** = **	** = **	** = **	**−**	**−**
**LARVA & ADULT**	Sex-Ratio	**+**	**+**	**+**	** = **	** = **	** = **
**ADULT**	Fecundity	** = **	**−**	** = **	**−**	**−**	** = **
	Adult female lifespan	**−**	** = **		**−**	**−**	
	Adult male lifespan	** = **	** = **		**−**	**−**	

*Drosophila melanogaster* lines were kept during one (F1) or ten generations (F10) on C18∶0- or C18∶1-rich food. F9 individuals kept on either fatty acid were transferred at the beginning of the 10^th^ generation on standard medium (F10-Std). The signs in the table represents the oviposition and fitness performance of exposed lines compared to controls (**−**: lower; +: higher; = : similar).

In contrast, C18∶1 exposure only induced negative effects specially on adult traits (fecundity, adult lifespan). These defects likely resulted of a direct – non-genetic – effect of C18∶1 since they disappeared in F10-Std individuals. This also indicates that lines could not adapt, even after 10 generations exposure, to C18∶1-rich food. However, C18∶1 exposure induced a persistent effect inF10 and F10-Std larvae which increased both their size and lapse time to initiate pupation. This suggests that C18∶1-rich diet has altered the genetic network integrating the interaction between the control of larval growth size and metamorphosis onset [59].

In summary, FA-conditioning specifically affected food oviposition preference and fitness according to the (1) substance used, (2) exposure period, and (3) number of exposed generations. Conditioning induced quick (F1) and/or persistent (F10-Std) changes. Such intertwined plasticity indicates that the genetic architecture underlying multiple behavioural and fitness traits are differently affected by sensory exposure. This stresses the importance, for future studies dealing with sensory and food adaptation, to examine the effect of the gene-environment interaction [3], [13], [60], [61], [62] both on short and long time scales. If *D. melanogaster* is capable of a similarly fast and durable adaptation to diverse novel food sources, this could explain the worldwide expansion of this generalist-diet species.

## Supporting Information

Figure S1 **Effect of transient exposure on fecundity and adult lifespan.** (**a**) Fecundity was measured in the progeny of female and male flies similarly exposed. The exposure and test conditions (Exposure/test) are indicated below the graphs (see legend of Figure 2). The daily egg production, measured between days 1 and 15 (corresponding to the female age), was cumulated with time. For the sake of clarity, the results obtained with C18∶0- and C18∶1-exposed flies are separately shown on left and right panel, respectively, whereas the control line (*) is shown on both panels. Egg production was simultaneously compared for all conditions, using a Kruskal-Wallis test (KW_10df_ = 67.26; *p*<0.0001; letters indicate significant differences at level *p* = 0.05; *N* = 17–20). (**b**) The box-and-whisker plots indicate the cumulative mortality after 22 days of adult life in females (left) and males (right). The conditions used for exposure and test are indicated below each plot. Differences in female and male mortality were separately assessed with Kruskal-Wallis tests (KW_6df_ = 29.30 and KW_6df_ = 40.08, respectively; both *p*<0.0001; letters indicate significant differences at level *p* = 0.05; *N* = 10). (**c**) Survival was measured in the progeny of female (left) and male (right) flies similarly exposed. Exposure and test conditions are indicated below the graphs (see legend of Fig. 2; *N* = 10).
(TIF)

Figure S2 **Effect of permanent exposure on preimaginal fitness.** Embryonic (**a**) and larval (**b**) survival was measured in lines permanently exposed to FA during one generation (F1), 10 generations (F10), or 9 generations+the 10^th^ generation on standard food (F10-Std). Exposure and test conditions are indicated below the graphs (see legend of Fig. 3). A slight effect was detected at both developmental stages using Kruskal-Wallis test (**a**: KW_6df_ = 14.09, *p* = 0.029; **b**: KW_6df_ = 14.56, *p* = 0.024; letters indicate significant differences at level *p* = 0.05; *N* = 10). (**c**) The overall size of female (left) and male (right) L3 increased in F10 and F10-Std C18∶1-exposed lines (KW_6df_ = 58.5 and KW_6df_ = 80.00, respectively; both *p*<0.0001; letters indicate significant differences; *N* = 18–35). The photographs above the plots show representative control and F10 C18∶1-exposed larvae. Scale bars = 3 mm.
(TIF)

Figure S3 **Effect of permanent exposure on adult lifespan.** Female (left) and male (right) adult survival was measured in lines permanently exposed to each FA during one generation (F1) or 10 generations (F10). Exposure and test conditions are indicated below the graphs (see legend of Fig. 3; *N* = 10).
(TIF)
