# Supplementary figures and images for: Transient and Permanent Experience with Fatty Acids Changes Drosophila melanogaster Preference and Fitness
Source: PLoS One. 2014 Mar 25;9(3):e92352. doi: 10.1371/journal.pone.0092352 (PMC3965419; doi:10.1371/journal.pone.0092352)

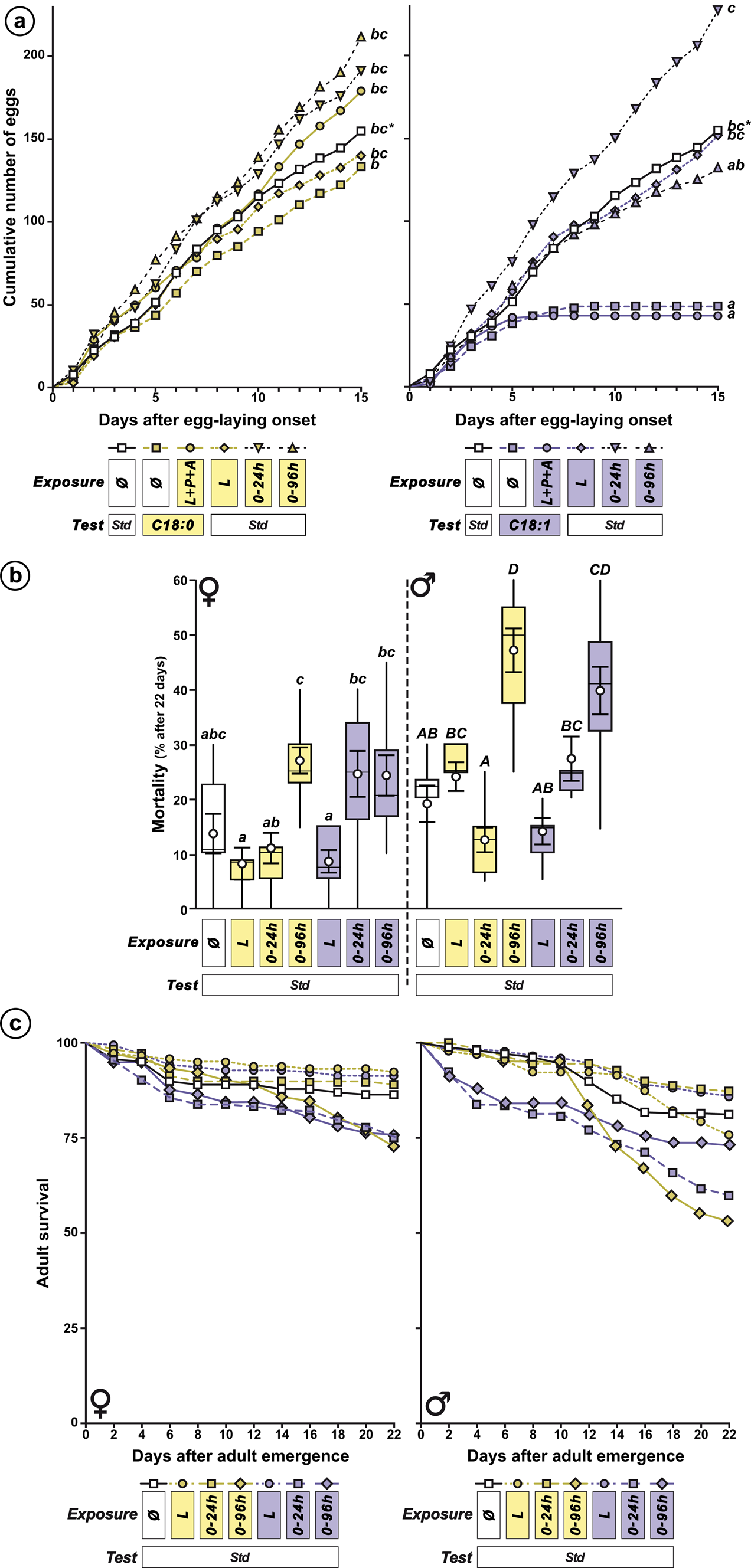

Supplement: Figure S1 — Effect of transient exposure on fecundity and adult lifespan. (a) Fecundity was measured in the progeny of female and male flies similarly exposed. The exposure and test conditions (Exposure/test) are indicated below the graphs (see legend of Figure 2). The daily egg production, measured between days 1 and 15 (corresponding to the female age), was cumulated with time. For the sake of clarity, the results obtained with C18∶0- and C18∶1-exposed flies are separately shown on left and right panel, respectively, whereas the control line (*) is shown on both panels. Egg production was simultaneously compared for all conditions, using a Kruskal-Wallis test (KW10df = 67.26; p<0.0001; letters indicate significant differences at level p = 0.05; N = 17–20). (b) The box-and-whisker plots indicate the cumulative mortality after 22 days of adult life in females (left) and males (right). The conditions used for exposure and test are indicated below each plot. Differences in female and male mortality were separately assessed with Kruskal-Wallis tests (KW6df = 29.30 and KW6df = 40.08, respectively; both p<0.0001; letters indicate significant differences at level p = 0.05; N = 10). (c) Survival was measured in the progeny of female (left) and male (right) flies similarly exposed. Exposure and test conditions are indicated below the graphs (see legend of Fig. 2; N = 10). (TIF) [file pone.0092352.s001.tif]

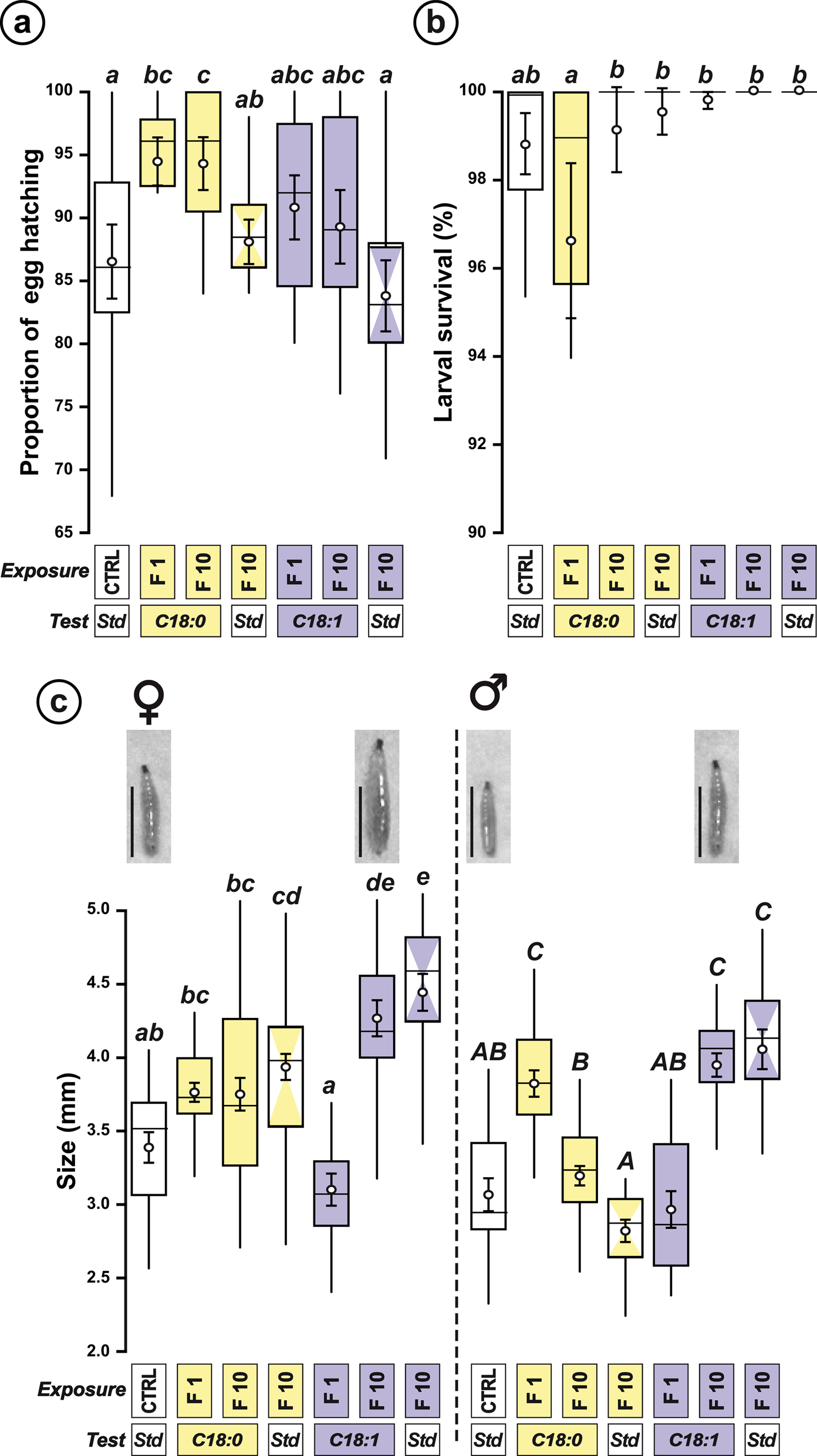

Supplement: Figure S2 — Effect of permanent exposure on preimaginal fitness. Embryonic (a) and larval (b) survival was measured in lines permanently exposed to FA during one generation (F1), 10 generations (F10), or 9 generations+the 10th generation on standard food (F10-Std). Exposure and test conditions are indicated below the graphs (see legend of Fig. 3). A slight effect was detected at both developmental stages using Kruskal-Wallis test (a: KW6df = 14.09, p = 0.029; b: KW6df = 14.56, p = 0.024; letters indicate significant differences at level p = 0.05; N = 10). (c) The overall size of female (left) and male (right) L3 increased in F10 and F10-Std C18∶1-exposed lines (KW6df = 58.5 and KW6df = 80.00, respectively; both p<0.0001; letters indicate significant differences; N = 18–35). The photographs above the plots show representative control and F10 C18∶1-exposed larvae. Scale bars = 3 mm. (TIF) [file pone.0092352.s002.tif]

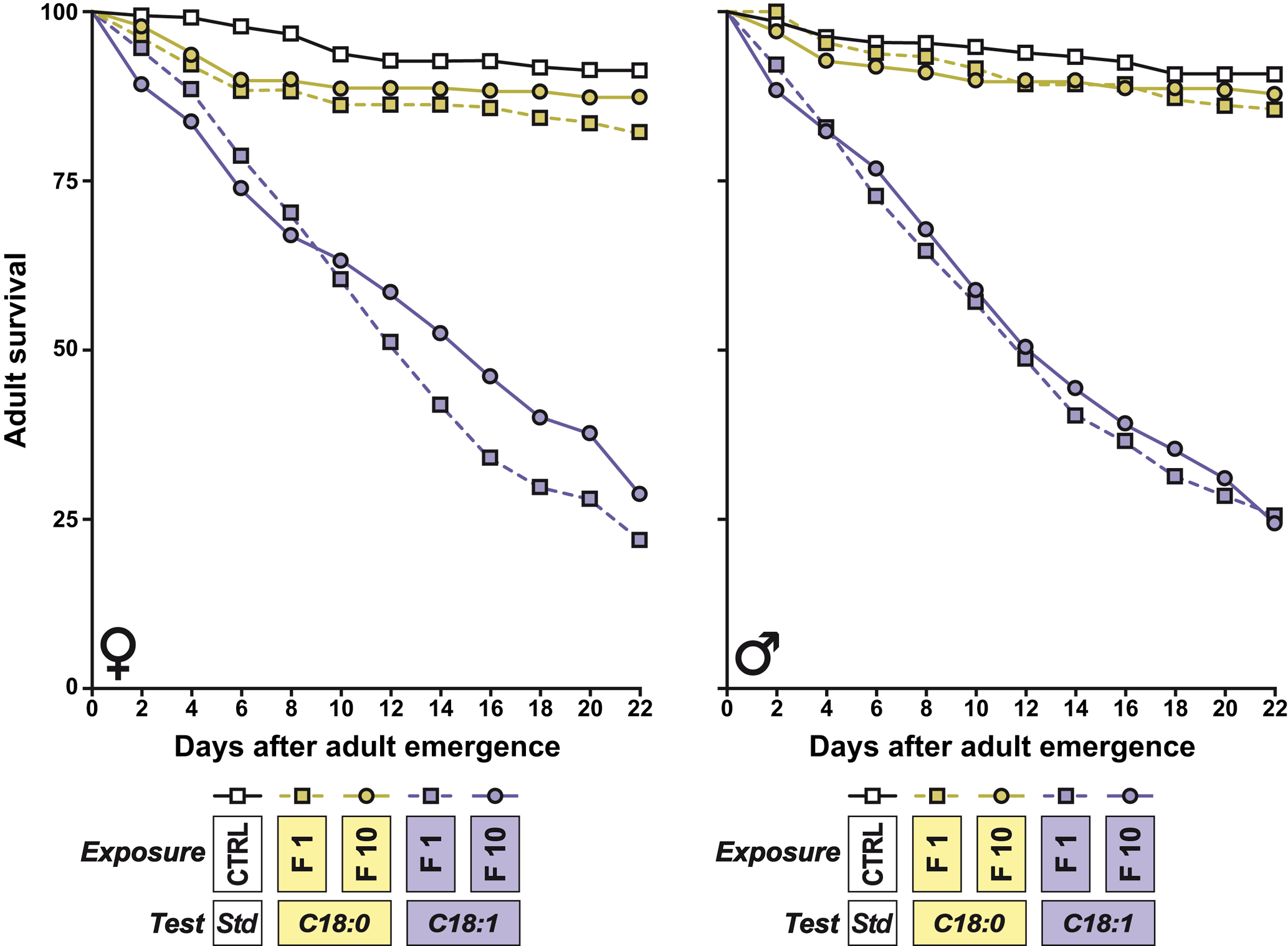

Supplement: Figure S3 — Effect of permanent exposure on adult lifespan. Female (left) and male (right) adult survival was measured in lines permanently exposed to each FA during one generation (F1) or 10 generations (F10). Exposure and test conditions are indicated below the graphs (see legend of Fig. 3; N = 10). (TIF) [file pone.0092352.s003.tif]
